# The *Alteromonas macleodii* ribosome enables consecutive incorporation of bulky D-amino acids into peptides

**DOI:** 10.1093/nar/gkag341

**Published:** 2026-04-21

**Authors:** Takayuki Katoh, Hiraku Takada, Maxwell Sigal, Hiroaki Suga

**Affiliations:** Department of Chemistry, Graduate School of Science, The University of Tokyo, 7-3-1 Hongo, Bunkyo-ku, Tokyo 113-0033, Japan; Department of Biotechnology, Faculty of Engineering, Toyama Prefectural University, 5180 Kurokawa, Imizu-shi, Toyama 939-0398, Japan; Department of Chemistry, Graduate School of Science, The University of Tokyo, 7-3-1 Hongo, Bunkyo-ku, Tokyo 113-0033, Japan; Department of Chemistry, Graduate School of Science, The University of Tokyo, 7-3-1 Hongo, Bunkyo-ku, Tokyo 113-0033, Japan

## Abstract

Genetic code reprogramming allows for ribosomal incorporation of exotic amino acids, such as d-α- and d-β-amino acids, into peptides. However, their incorporation efficiency remains much lower than that of canonical l-amino acids, making their multiple/consecutive incorporations difficult. The side chain of d-α-amino acids clashes with U2506 of 23S rRNA, hindering the incorporation of d-α-amino acids with bulky side chains; therefore, consecutive incorporation has been limited to small d-α-amino acids. To overcome this limitation, we screened ribosomes from phylogenetically diverse bacterial species to identify variants that enable consecutive incorporation of bulky d-amino acids. The *Alteromonas macleodii* (AM) ribosome is capable of incorporating d-α-, d-β-, and α,α-disubstituted amino acids with efficiencies superior to the *Escherichia coli* (EC) ribosome. It elongates six consecutive d-Ser and two consecutive 1-aminocyclobutane-1-carboxylic acid with yields 6.1- and 7.3-fold higher than the EC ribosome. Moreover, consecutive incorporation of nine types of bulky d-α-amino acids (d-Asn, d-Asp, d-Gln, d-Met, d-Phe, d-Thr, d-Trp, d-Tyr, d-Val) was achieved for the first time. A model macrocyclic peptide containing four d-amino acids and one α,α-disubstituted amino acid was also synthesized by the AM ribosome. These results expand the potential of ribosomal synthesis for peptide libraries containing structurally diverse, bulky d-amino acids.

## Graphical Abstract

**Graphical Abstract:**
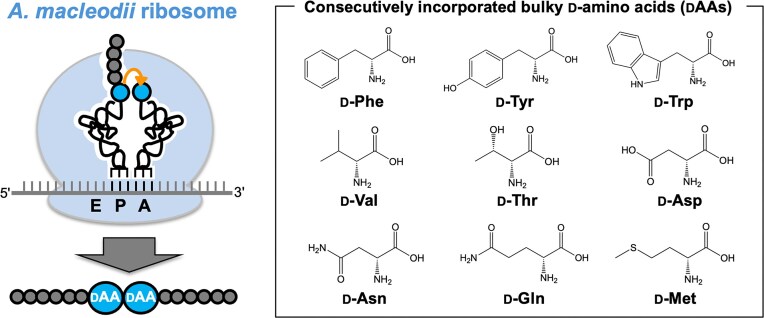

## Introduction

In nature, the ribosome exclusively utilizes canonical l-α-amino acids for protein and peptide translation. However, advances in genetic code reprogramming methodologies—including stop codon suppression [1], sense codon reassignment [2], and quadruplet codon usage [3]—have enabled the ribosomal incorporation of various nonproteinogenic amino acids (npAAs). These encompass not only l-α-amino acids with modified side chains but also backbone-altering npAAs such as d-α-amino acids, α,α-disubstituted α-amino acids, *N*-methyl/alkyl-α-amino acids, β-amino acids, and γ-amino acids [4–14]. Generally, the incorporation of backbone-altering npAAs by the ribosome is much less efficient than that of l-α-amino acids, primarily due to: 1) inefficient accommodation of aminoacyl-tRNA (npAA-tRNA) at the ribosomal A-site caused by low binding affinity to EF-Tu [6, 15, 16]; 2) slow peptidyl transfer between the P-site peptidyl-tRNA and A-site aminoacyl-tRNA [7, 17–19]; and 3) ribosomal stalling triggered by interactions between the npAA-containing nascent peptide and the ribosomal exit tunnel [20]. To address the first two issues, we previously developed an engineered tRNA, tRNA^Pro1E2^, featuring specific T-stem and D-arm motifs that effectively recruit EF-Tu and EF-P to enhance aminoacyl-tRNA accommodation and peptidyl transfer, respectively (Supplementary Fig. S1A) [7]. Notably, while EF-P naturally promotes peptidyl transfer between proline residues [21, 22], we demonstrated that it can also facilitate the incorporation of backbone-altering npAAs when they are charged onto tRNA^Pro1E2^, which possesses the specific D-arm motif recognized by EF-P [7]. Regarding the third issue, ATP-binding cassette family-F (ABC-F) proteins—including EttA, Uup, YbiT, and YhsS—can be utilized [20, 23]. These E-site-binding ATPases possess an interdomain linker, located between two ATP-binding domains, that inserts into the peptidyl transferase center (PTase center, PTC), inducing rearrangements of the PTC and P-site peptidyl-tRNA to rescue stalled ribosomes [24–29]. We recently reported that these ABC-F proteins enhance the incorporation of diverse backbone-altering npAAs, such as d-α-amino acids, *N*-methyl-α-amino acids, and β-amino acids [20].

**Figure 1.**
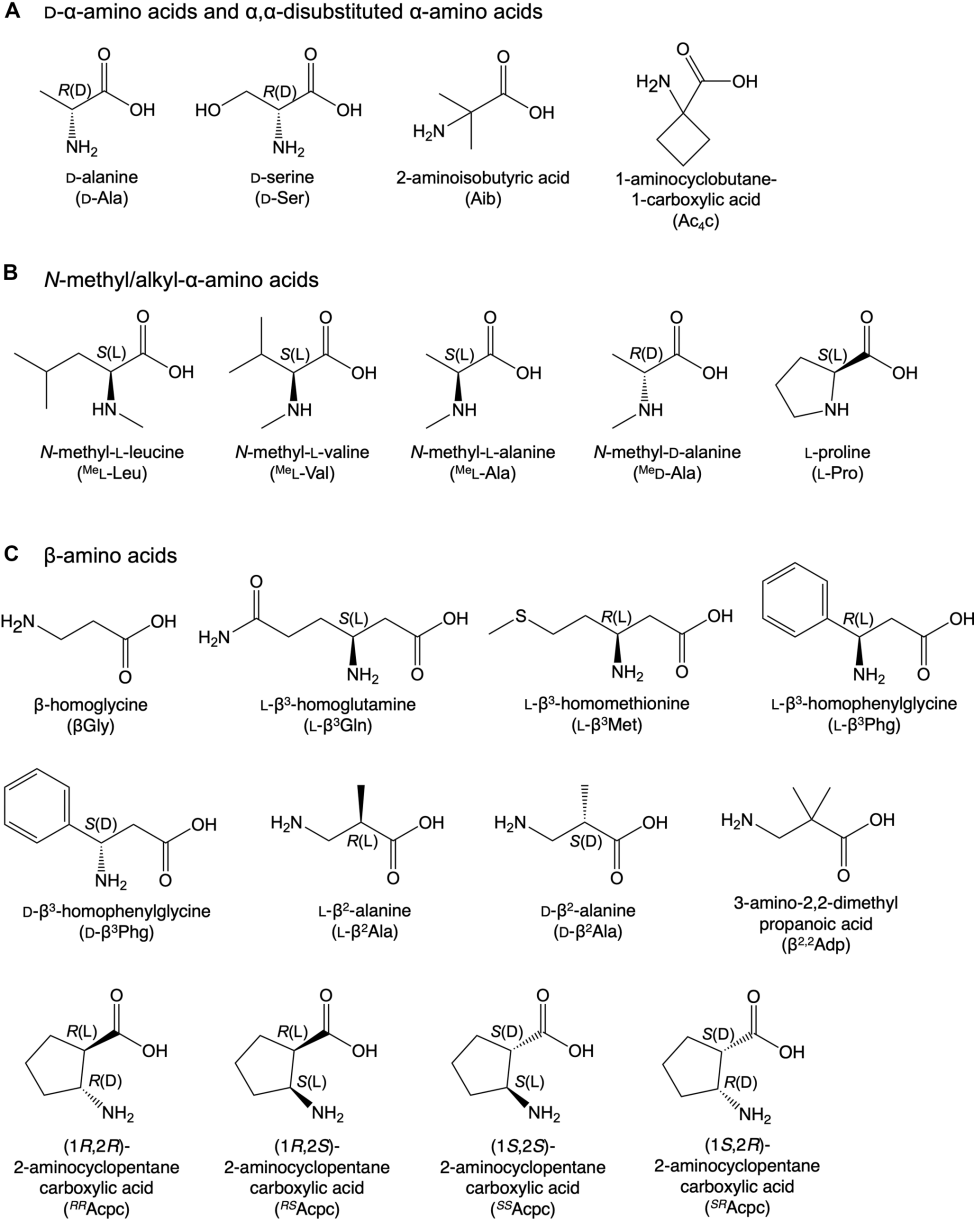
Chemical structures of nonproteinogenic amino acids evaluated for ribosomal incorporation. (**A**) d-α-amino acids and α,α-disubstituted α-amino acids; (**B**) *N*-methyl/alkyl-α-amino acids; and (**C**) β-amino acids. Chiralities of stereocenter carbon atoms are indicated as *R*/*S* and l/d configurations.

However, despite these advancements, the consecutive incorporation of backbone-altering npAAs remains a formidable challenge [6]. This process requires the peptidyl transfer reaction to proceed while both the P- and A-sites are occupied by less reactive npAAs. Regarding d-amino acids specifically, Englander *et al.* proposed that nucleotides A2058, A2059, and A2062 in domain V of the 23S rRNA render the P-site peptidyl-d-Phe-tRNA inactive [30]. In the presence of peptidyl-d-Phe-tRNA, conformational changes of these nucleotide residues prevent the ribosome from correctly positioning the P-site peptidyl-d-Phe-tRNA and the A-site aminoacyl-tRNA for the PTase reaction. Melnicov *et al.* suggested that when d-Phe-tRNA occupies the A site, its side chain sterically clashes with the universally conserved nucleotide U2506, thus highlighting U2506’s role in excluding d-amino acids during translation [19]. Consequently, consecutive incorporation has been limited to d-amino acids with relatively small side chains, such as d-Ala and d-Ser (Fig. 1A) [6]; Bulkier d-amino acids remain difficult to incorporate consecutively due to these steric hindrances. Similarly, among α,α-disubstituted amino acids, only those with relatively small side chains—2-aminoisobutyric acid (Aib) and 1-aminocyclobutane-1-carboxylic acid (Ac_4_c)—have been successfully incorporated consecutively (Fig. 1A) [7, 8]. To our knowledge, other types of α,α-disubstituted amino acids have not been demonstrated for consecutive incorporation. These limitations suggest that translating peptides or proteins composed primarily or entirely of d-amino acids and/or α,α-disubstituted amino acids is unfeasible with current strategies. Achieving this would require ribosome variants that bypass steric clashes and more efficiently incorporate d-amino acids than the *Escherichia coli* (EC) ribosome—the *de facto* standard for *in vitro* translation. While we have been utilizing an EC-based reconstituted *in vitro* translation system, termed the Flexible *In vitro* Translation (FIT) system [31], for backbone-altering npAA incorporation, including d-amino acids, the EC ribosome may not be optimal for this purpose. To date, however, ribosomes from other organisms have not been evaluated for d-amino acid incorporation. Therefore, we screened phylogenetically diverse bacterial species to identify ribosomes capable of superior d-amino acid incorporation, aiming to enable the consecutive incorporation of diverse, including bulky, d-amino acids.

## Materials and methods

### Preparation of bacterial ribosomes

*E. coli* strain MRE600 and *Bacillus subtilis* strain wt168 were grown at 37°C in LB medium until mid-log phase. *Alteromonas macleodii* ATCC 27126 strain was grown at 30°C in LBN medium (1% peptone, 0.5% yeast extract, 2.4% NaCl) supplemented with 10 mM MgSO₄ until mid-log phase. *Paraburkholderia largidicola* KT205 strain was grown at 30°C in TSB medium (1.7% casein peptone, 0.3% soybean peptone, 0.5% NaCl, 0.25% K₂HPO₄, 0.25% glucose) until mid-log phase. *Caulobacter crescentus* NA1000 strain was grown at 30°C in PYE medium (0.2% peptone, 0.1% yeast extract, 0.02% MgSO₄·7H₂O, 0.01% CaCl₂) until mid-log phase. Cells were harvested by centrifugation and stored as pellets at -80°C. Pellets were thawed in suspension buffer [10 mM HEPES-KOH (pH 7.6), 50 mM KCl, 10 mM Mg(OAc)₂, 7 mM 2-mercaptoethanol] and disrupted either by passage through a microfluidizer LV1 (Microfluidics) for *E. coli, B. subtilis, P. largidicola*, and *C. crescentus*, or by snap-freezing in liquid nitrogen followed by pulverization using a Multi-Beads Shocker instrument (Yasui Kikai) for *A. macleodii*. For all species, crude lysates were clarified by centrifugation (4°C, 10 000 × g, 30 min). The clarified lysates were then mixed with an equal volume of suspension buffer containing 3 M ammonium sulfate and incubated on ice for 30 min. Cell debris and precipitated proteins were removed by centrifugation (4°C, 10 000 × g, 30 min). The supernatant was applied to a custom-packed XK 26/20 column (Cytiva) packed with 20 ml Butyl Sepharose Fast Flow resin (Cytiva) equilibrated with buffer A [20 mM HEPES-KOH (pH 7.6), 1.5 M (NH₄)₂SO₄, 10 mM Mg(OAc)₂, 7 mM β-mercaptoethanol]. The column was washed with 40 ml of buffer B [20 mM HEPES-KOH (pH 7.6), 10 mM Mg(OAc)₂, 7 mM β-mercaptoethanol] containing 1.2 M ammonium sulfate, and proteins were eluted with a 1.2 M to 0 M ammonium sulfate gradient in buffer B. Fractions containing ribosomes were pooled and layered onto an equal volume of 30% sucrose cushion buffer [20 mM HEPES-KOH (pH 7.6), 10 mM Mg(OAc)₂, 30 mM NH₄Cl, 30% sucrose, 7 mM 2-mercaptoethanol] and ultracentrifuged (4°C, 100 000 × g, 16 h). Pellets were resuspended in HEPES:Polymix buffer [20 mM HEPES-KOH (pH 7.5), 2 mM DTT, 15 mM Mg(OAc)₂, 95 mM KCl, 5 mM NH₄Cl, 0.5 mM CaCl₂, 8 mM putrescine, 1 mM spermidine] and stored at − 80°C until use for *in vitro* translation assays.

### Preparation of mRNAs, tRNAs, and flexizymes

Sequences for mRNAs, tRNAs, and flexizymes used in this study are listed in Supplementary Table S1. Template DNAs for RNA transcription were generated via extension and PCR with specific forward and reverse primers, as detailed in Supplementary Table S1. PCR products were purified using phenol/chloroform extraction followed by ethanol precipitation. Transcription of tRNAs was performed at 37°C for 16 h in a mixture containing 40 mM Tris-HCl (pH 8.0), 3.75 mM NTP mix, 5 mM guanosine monophosphate (GMP), 22.5 mM MgCl_2_, 1 mM dithiothreitol, 1 mM spermidine, 0.01% Triton X-100, 0.04 U/µL RNasin RNase inhibitor (Promega), and 0.12 µM T7 RNA polymerase. For flexizyme transcription, NTP was increased to 5 mM, and GMP was omitted. Transcribed RNAs were incubated with RQ1 DNase (Promega) for 30 min at 37°C, recovered via isopropanol precipitation, and purified by PAGE (8% for tRNAs, 12% for flexizymes) containing 6 M urea. DNAs for mRNA transcription were directly added to the FIT system for coupled transcription/translation reactions.

### Preparation of aminoacyl-tRNA

npAAs and l-Ala were preactivated as 3,5-dinitrobenzyl ester (DBE), *p*-chlorobenzyl thioester (CBT), or cyanomethyl ester (CME) as described [5]. d-Val, d-Thr, and d-Ile were activated as CBT; d-Phe, d-Tyr, d-Trp, and *N*-chloroacetyl-d-tyrosine (^ClAc^d-Tyr) as CME; and other amino acids as DBE. Aminoacylation was performed at 0°C using 50 mM HEPES-KOH (pH 7.5) or Bicine-KOH (pH 9.0), 200 mM MgCl_2_, 20% DMSO, 25 µM dFx or eFx, 25 µM tRNA, and 5 mM activated amino acid. dFx was used for DBE forms; eFx for CBT and CME (with MgCl_2_ increased to 600 mM). Reaction conditions are summarized in Supplementary Table S2. Resulting aminoacyl-tRNAs were ethanol-precipitated, washed with 70% ethanol, and dissolved in 1 mM sodium acetate (pH 5.2).

### Preparation of *A. macleodii* EF-P and Uup

*A. macleodii* (NCBI Reference Sequence: NC_018632.1, ATCC 27126) EF-P (NCBI Reference Sequence: WP_014948217.1) was codon optimized for *E. coli* K12 and cloned into a custom pET28a vector, which contained an HRV3C cleavage site instead of thrombin. For the production of modified AM EF-P, an additional pETDuet-1 vector was used, which contained *E. coli* EpmA, EpmB, and EpmC genes. These two plasmids were co-transformed into *E. coli* Rosetta2(DE3)pLysS (Novagen) and inoculated into 1 L LB media supplemented with 100 µg/mL ampicillin, 50 µg/mL kanamycin, and 30 µg/mL chloramphenicol. Cultures were grown at 37°C until OD_600_ reached 0.5 – 0.6, after which 0.5 mM IPTG was added. The cultures were incubated for an additional 21 h at 18°C. Cells were harvested by centrifugation at 7000 g, 4°C for 10 min and resuspended in 100 mL buffer A [20 mM Tris-HCl (pH 7.5), 150 mM NaCl, 5 mM imidazole, 1 mM β-mercaptoethanol] supplemented with 0.1 mM PMSF. *E. coli* were lysed by sonication, the lysate was clarified by centrifugation at 15 000 g, 4°C for 20 min, and the supernatant was loaded onto a gravity column packed with 4 mL TALON® Metal Affinity Resin (Takara Bio) equilibrated with buffer A. The protein was eluted in 5 mL buffer B [20 mM Tris HCl (pH 7.5), 150 mM NaCl, 300 mM imidazole, 1 mM β-mercaptoethanol] and desalted using PD-10 desalting columns (Cytiva, 17 085 101) to buffer C [20 mM Tris HCl (pH 7.5), 150 mM NaCl, 1 mM β-mercaptoethanol]. The *N*-terminal polyhistidine tag of EF-P was cleaved using Turbo3C protease (Accelagen) at 4°C for 18 h. Afterwards, the protein was reloaded onto a gravity column packed with 4 mL TALON® Metal Affinity Resin equilibrated with buffer C. The flow-through and subsequent 2 mL wash fraction were pooled, concentrated using an Amicon Ultra-4 10 kDa cutoff (Merck Millipore), and filtered with a 0.45 µm filter.

AM Uup gene was cloned into a pET28a-TEV vector and transformed into *E. coli* BL21 (DE3) cells. Cultures were grown in LB medium with 0.5 mM IPTG for 3 h at 37°C and lysed by sonication. The lysate was loaded onto a His-TALON crude (5 mL) column (Cytiva) and washed with buffer D [20 mM Tris-HCl (pH 8.0), 200 mM NaCl, 1 mM dithiothreitol] containing 10 mM imidazole. His-tagged AM Uup was then eluted by increasing imidazole concentration up to 300 mM in buffer D. The eluted protein was concentrated using an Amicon Ultra Centrifugal Filter (Merck Millipore), and the buffer was exchanged for an imidazole-free buffer D.

### *In vitro* translation of model peptides

Translation reactions were carried out at 37°C for 30 min using the reconstituted Flexible *In vitro* Translation (FIT) system, with details listed in Supplementary Table S3. For peptide quantification, 0.05 mM [^14^C]-Asp was substituted for cold Asp in the translation mix. Reactions were stopped by adding an equal volume of stop solution [0.9 M Tris-HCl (pH 8.45), 8% SDS, 30% glycerol, 0.001% xylene cyanol] and heating at 95°C for 2 min. Peptides were separated by 15% tricine SDS-PAGE and visualized by autoradiography (Typhoon FLA 7000, Cytiva). Absolute peptide yields were determined based on radioisotope intensity relative to total [^14^C]-Asp in the reaction.

For peptide identification by MALDI-TOF mass spectrometry (MS), translation was performed using cold Asp. Following translation, 2.5 µL of the reaction mixture was diluted with an equal volume of 2 × TBS buffer [100 mM Tris-HCl (pH 7.6), 300 mM NaCl] and incubated with anti-FLAG M2 affinity gel (Sigma-Aldrich) for 15 min at room temperature. The beads were washed twice with 1 × TBS buffer [50 mM Tris-HCl (pH 7.6), 150 mM NaCl] and then eluted with 0.1% trifluoroacetic acid. The eluted peptides were purified using SPE C-tip (Nikkyo Technos) and eluted with 0.85 μL of 80% acetonitrile/0.5% acetic acid containing 50%-saturated (*R*)-cyano-4-hydroxycinnamic acid. Samples were analyzed on an UltrafleXtreme (Bruker Daltonics) in reflector/positive mode, using peptide calibration standard II (Bruker Daltonics) for external mass calibration.

## Results

### Translation of model peptides containing consecutive npAAs by means of bacterial ribosomes

**Figure 2.**
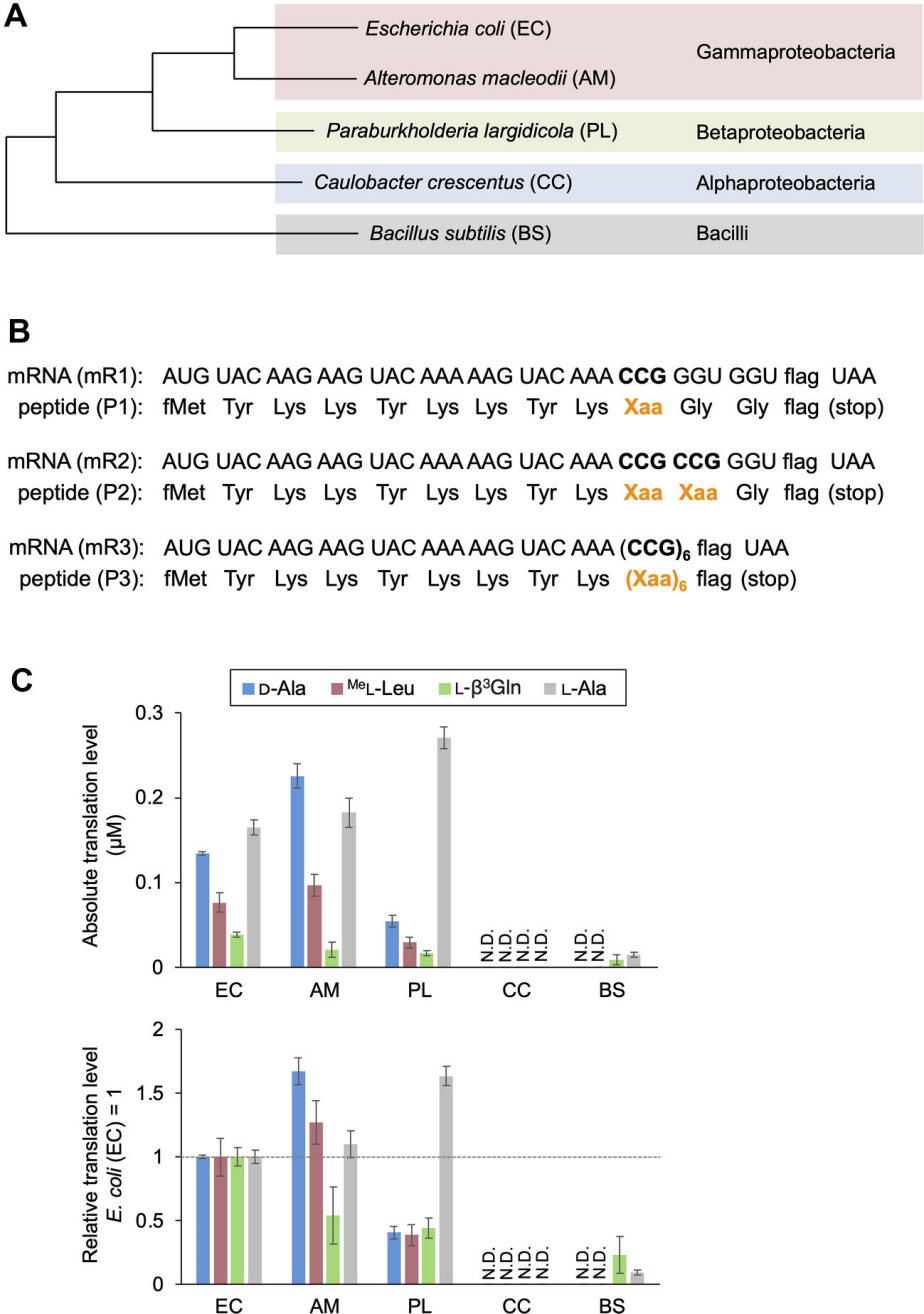
Translation of model peptides containing npAAs using ribosomes from phylogenetically diverse bacteria. (**A**) Phylogenetic tree of six bacterial species included in this study, illustrating evolutionary divergence based on their 23S rRNAs. (**B**) Sequences of mRNAs (mR1–3) and corresponding peptides (P1–3) used in this study. “Xaa” denotes arbitrary npAAs incorporated at CCG codons using precharged npAA-tRNA^Pro1E2^. The “flag” sequence consists of Asp–Tyr–Lys–Asp–Asp–Asp–Asp–Lys. (**C**) Quantification of absolute (top) and relative (bottom) translation levels for P2 peptides containing npAAs. d-Ala, ^Me^l-Leu, l-β^3^Gln, and l-Ala were incorporated at two consecutive CCG codons of mR2. Translations utilized ribosomes from *E. coli* (EC), *Alteromonas macleodii* (AM), *Paraburkholderia largidicola* (PL), *Caulobacter crescentus* (CC), and *Bacillus subtilis* (BS) with the *E. coli* translation factors, aminoacyl-tRNA synthetases, tRNAs, and other necessary factors. See Supplementary Table S3 for translation conditions. Relative translation levels are normalized to those obtained with *E. coli* ribosomes (set as 1). Error bars represent standard deviations from three independent experiments. Raw Tricine SDS-PAGE results are shown in Supplementary Fig. S2.

**Figure 3.**
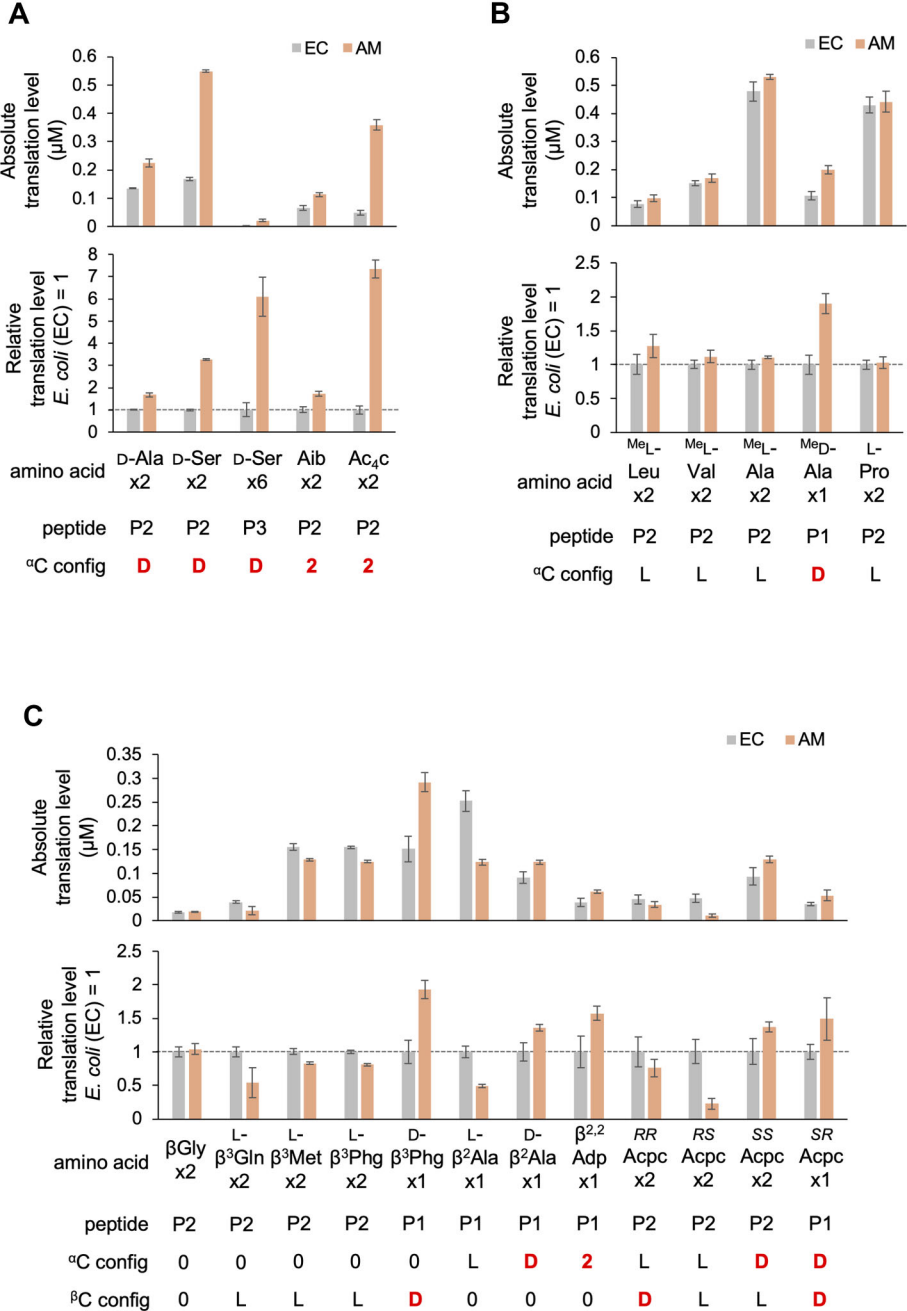
Translation of model peptides containing npAAs using *E. coli* and *A. macleodii* ribosomes. (**A**–**C**) Quantification of absolute (top) and relative (bottom) translation levels of P1, P2, or P3 peptides containing npAAs. d-α-amino acids or α,α-disubstituted α-amino acids (**A**), *N*-methyl/alkyl-α-amino acids (**B**), and β-amino acids (**C**) were incorporated. Peptide names and the configurations of α- and β-carbons are indicated at the bottom, where “0” denotes no substituents and “2” denotes dialkyl substituents. Translation was performed using *E. coli* (EC) or *A. macleodii* (AM) ribosomes, alongside *E. coli* translation factors, aminoacyl-tRNA synthetases, tRNAs, and other required components. See Supplementary Table S3 for detailed translation conditions. Relative translation levels are normalized to those of the *E. coli* ribosome (set as 1). Error bars indicate standard deviations from three independent experiments. Raw tricine SDS-PAGE results are in Supplementary Fig. S2.

We selected five bacterial species—*Alteromonas macleodii* (AM), *Paraburkholderia largidicola* (PL), *Caulobacter crescentus* (CC), *Bacillus subtilis* (BS), and *Escherichia coli* (EC)—based on phylogenetic diversity and the ease of culture and ribosome preparation (Fig. 2A). These species span distinct bacterial lineages (Alpha–, Beta–, and Gammaproteobacteria, and Bacilli) and include both Gram–negative and Gram–positive organisms. Using these ribosomes, we performed *in vitro* translation of model peptides incorporating three classes of npAAs: [1] d-α- and α,α-disubstituted amino acids (Fig. 1A), [2] *N*-methyl- and *N*-alkyl-α-amino acids (Fig. 1B), and [3] β-amino acids (Fig. 1C). For the initial screening, d-alanine (d-Ala), *N*-methyl-l-leucine (^Me^l-Leu), and l-β^3^-homoglutamine (l-β^3^Gln) were selected, along with l-Ala as a control. These amino acids were precharged onto tRNA^Pro1E2^_CGG_ (Supplementary Fig. S1A) using a flexizyme variant, dFx [32, 33], and incorporated at two consecutive CCG codons of mRNA (mR2) to yield peptide P2 (Fig. 2B). Translation was performed using customized, heterologous FIT systems where all components were derived from EC and were identical to the original FIT system, except that the EC ribosome was replaced by ribosomes from AM, PL, CC, or BS. (see Supplementary Table S2 for details). To enhance npAA incorporation, 5 µM EC EF-P and 1 µM EC Uup were also added to the reactions.

To estimate translation levels, peptides were labeled with [^14^C]-Asp, separated via Tricine SDS-PAGE, and quantified by autoradiography. [^14^C]-Asp was incorporated into the C-terminal FLAG-tag (DYKDDDDK), which contains multiple Asp residues, allowing for sensitive detection of the translated peptides. The absolute translation levels for P2-d-Ala, P2-^Me^l-Leu, P2-l-β^3^Gln, and P2-l-Ala using the EC ribosome were 0.13, 0.08, 0.04, and 0.17 µM, respectively (Fig. 2C top, Supplementary Fig. S2A). Translation levels using other ribosomes were normalized to the EC ribosome (set as 1, Fig. 2C bottom). The relative P2-l-Ala translation level with the PL ribosome was 1.6, indicating that it is 1.6-fold more active than the EC ribosome for l-Ala incorporation when combined with EC translation factors. The AM ribosome showed activity similar to that of the EC ribosome (1.1-fold). In contrast, the relative P2-l-Ala levels for CC and BS were 0 and 0.1, respectively. This 10% activity for the BS ribosome is consistent with a previous report by Chiba *et al.*, who demonstrated that a hybrid translation system utilizing BS ribosomes and EC translation factors yielded GFP at a level roughly 10–20% of that achieved with the all-EC system [34]. These results collectively suggest that while the BS ribosome is functionally compatible with the EC translation machinery to a limited extent, its efficiency is significantly reduced, likely due to its greater phylogenetic distance from EC compared to the AM and PL ribosomes.

Regarding npAA incorporation, the PL ribosome exhibited significantly lower translation levels for P2-d-Ala, P2-^Me^l-Leu, and P2-l-β^3^Gln than the EC ribosome (Fig. 2C bottom, each 0.4). In contrast, the AM ribosome showed higher levels for P2-d-Ala and P2-^Me^l-Leu (1.7 and 1.3, respectively) but a lower level for P2-l-β^3^Gln (0.5). These results indicate that the PL ribosome more strictly excludes these npAAs during translation compared to the EC ribosome, whereas the AM ribosome is more permissive for d-Ala and ^Me^l-Leu, but not for l-β^3^Gln. Peptide identities were verified by MALDI-TOF MS, confirming the production of the desired peptides without misincorporation (Supplementary Figs S3, S4, S5).

### Ribosomal incorporation of diverse npAAs using the *A. macleodii* ribosome

Encouraged by the initial screening, we further assessed the capability of the AM ribosome to incorporate a broader range of npAAs. Based on our findings, d-amino acids and α,α-disubstituted amino acids appeared to be particularly promising substrates for the AM ribosome. As representatives, we selected d-serine (d-Ser), Aib, and Ac_4_c, in addition to d-Ala (Fig. 1A). Two consecutive incorporations of d-Ala, d-Ser, Aib, and Ac_4_c into P2 using the AM ribosome yielded translation levels 1.7-, 3.3-, 1.7-, and 7.3-fold higher than those of the EC ribosome, respectively (Fig. 3A, Supplementary Fig. S2B). These results suggest that the AM ribosome exhibits a more pronounced advantage over the EC ribosome as the bulkiness of the d-amino acid (d-Ser > d-Ala) or α,α-disubstituted amino acid (Ac_4_c > Aib) increases. Furthermore, we achieved six consecutive incorporations of d-Ser into peptide P3, where the AM ribosome showed a 6.1-fold higher yield than the EC ribosome, indicating that increasing the number of consecutive d-amino acids accentuates the efficiency gap between the two ribosomes.

Next, we examined the consecutive incorporation of two *N*-methyl/alkyl-l-α-amino acids into P2. Translation of P2-^Me^l-Val, P2-^Me^l-Ala, and P2-l-Pro resulted in relative translation levels of 1.1, 1.1, and 1.0, respectively (Fig. 3B, Supplementary Fig. S2C). We also evaluated the single incorporation of ^Me^d-Ala into P1, as its incorporation was found to be too inefficient for consecutive introduction into P2. Notably, the translation of P1-^Me^d-Ala using the AM ribosome resulted in a 1.9-fold higher translation level than with the EC ribosome. These findings indicate that while *N*-methylation/alkylation of α-amino acids does not significantly differentiate the two ribosomes, their d-configuration is more readily accommodated by the AM ribosome.

Finally, we investigated the incorporation of both d-β-amino acids and l-β-amino acids. First, two consecutive insertions of β-homoglycine (βGly), which lacks a side chain, into P2 yielded similar translation levels for both the AM and EC ribosomes (Fig. 3C, Supplementary Fig. S2D). For β^3^-amino acids with l-configured side chains—namely l-β^3^Gln, l-β^3^-homomethionine (l-β^3^Met), and l-β^3^-homophenylglycine (l-β^3^Phg)—the AM ribosome showed lower efficiency than the EC ribosome, with relative yields of 0.5-, 0.8-, and 0.8-fold, respectively. In contrast, the insertion of d-β^3^Phg into P1 resulted in a 1.9-fold higher translation level with the AM ribosome. Similarly, for β^2^-amino acids, l-β^2^-homoalanine (l-β^2^Ala) was less efficiently incorporated into P1 with the AM ribosome (0.5-fold), whereas d-β^2^Ala and 3-amino-2,2-dimethylpropanoic acid (β^2,2^Adp) were enhanced by 1.4-fold and 1.6-fold, respectively. We also evaluated ribosomal incorporation of cyclic β^2,3^-amino acids using the four stereoisomers of 2-aminocyclopentanecarboxylic acid (*^RR^*Acpc, *^RS^*Acpc, *^SS^*Acpc, *^SR^*Acpc). As expected, *^RS^*Acpc (l-configurations at both α- and β-carbons) was inefficiently incorporated by the AM ribosome (0.2-fold), while *^SR^*Acpc (d-configurations at both carbons) showed the highest AM/EC ratio (1.5). The mixed-configuration isomers, *^RR^*Acpc and *^SS^*Acpc, showed intermediate values (0.8 and 1.4). In summary, the AM ribosome outperforms the EC ribosome in incorporating β-amino acids with d-configured side chains at either or both the α- and β-carbons, but is less effective for those with l-configurations. MALDI-TOF MS confirmed that all model peptides depicted in Fig. 3 were translated accurately without byproducts with both ribosomes (Supplementary Figs S3, S4).

### Replacement of *E. coli* EF-P and Uup with *A. macleodii* orthologs

**Figure 4.**
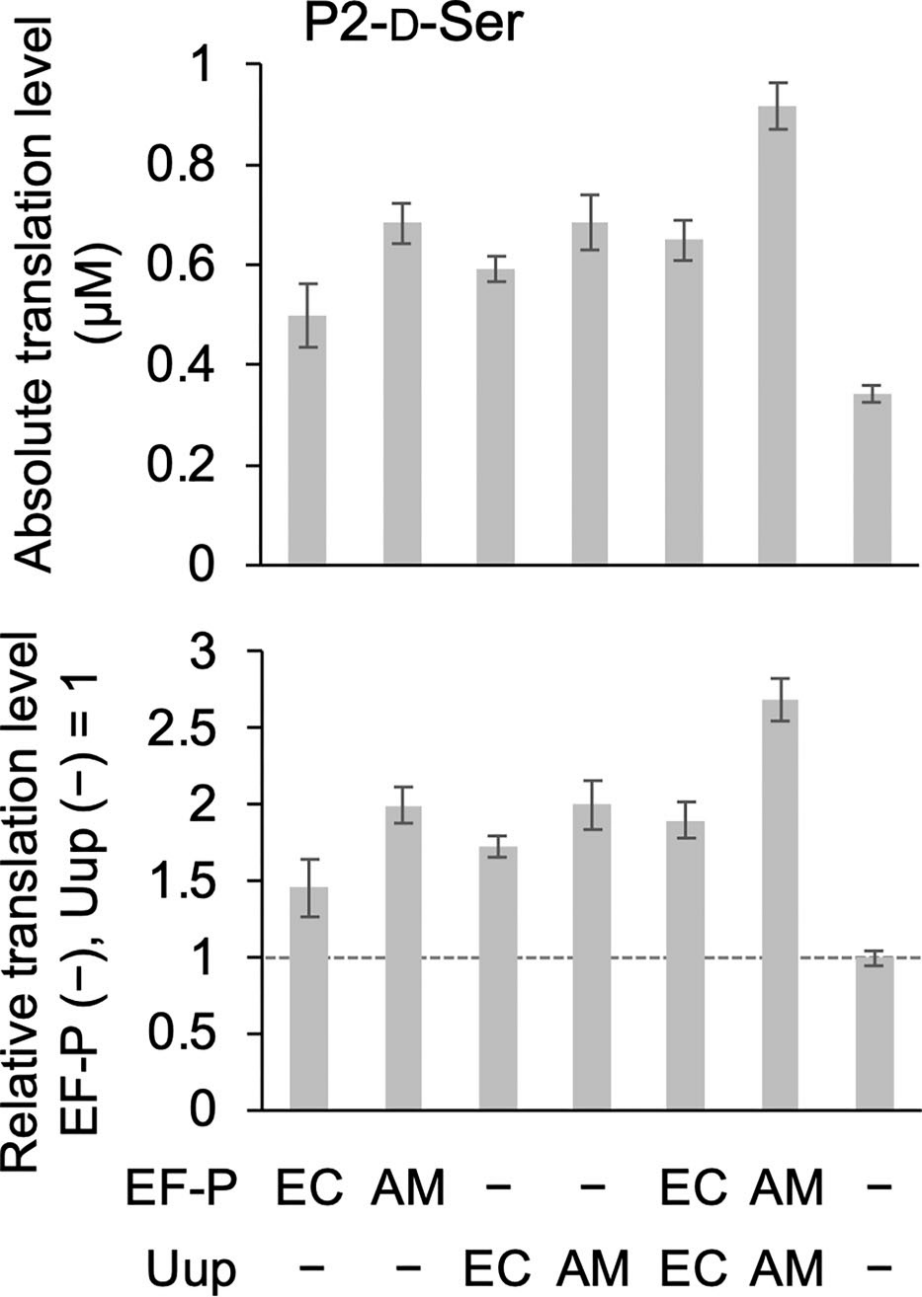
Translation of P2-d-Ser in the presence of *E. coli* or *A. macleodii* EF-P and Uup. Quantification of absolute (top) and relative (bottom) translation levels for P2 containing two consecutive d-Ser residues. Translation was performed using *A. macleodii* (AM) ribosomes with translation factors from *E. coli* (EC), except for EF-P and Uup. The translation system included 5 µM EF-P and 1 µM Uup. See Supplementary Table S3 for translation conditions. Error bars represent standard deviations from three independent experiments. Supplementary Fig. S2 provides raw Tricine SDS-PAGE results.

Since the backbone-altering npAAs examined in this study are generally inefficient substrates for translation, we supplemented the translation system with EF-P and Uup to promote their incorporation. EF-P promotes peptidyl transfer (especially for Pro and backbone-altering npAAs), and Uup is an ABC-F ATPase that rescues stalled ribosomes by remodeling the PTC. In the preceding experiments, these factors were derived from EC; however, we hypothesized that pairing the AM ribosome with EC EF-P and Uup might not be optimal. To test this, we evaluated the effects of AM EF-P and AM Uup in combination with the AM ribosome for the translation of P2-d-Ser (Fig. 4, Supplementary Fig. S2E). The addition of 5 µM EC EF-P or AM EF-P enhanced translation levels by 1.5-fold and 2.0-fold, respectively, compared to controls without EF-P. Similarly, supplementation with 1 µM EC Uup or AM Uup increased translation by 1.7-fold and 2.0-fold. Most notably, the combination of AM EF-P and AM Uup resulted in a 2.7-fold enhancement, whereas the corresponding EC combination yielded only a 1.9-fold increase. These results demonstrate that AM EF-P and AM Uup provide significantly greater synergy with the AM ribosome than their EC counterparts.

Both AM and EC EF-P proteins possess the conserved KPGKGQ modification motif (Supplementary Fig. S6A, B), which is characteristic of EF-P proteins that undergo β-lysylation and hydroxylation at the K34 residue [35]. Furthermore, orthologs of EpmA, EpmB, and EpmC—the enzymes responsible for these modifications—are conserved within the AM genome. This suggests that AM EF-P is naturally β-lysylated and hydroxylated in its native host, much like its EC counterpart. The AM EF-P used in this study was purified from EC cells after co-overexpression with the EC modification enzymes EpmA, EpmB, and EpmC (see Materials and Methods). Mass spectrometry analysis revealed that its K34 residue was only ∼50% β-lysylated (Supplementary Fig. S6C, D), likely due to the limited activity of the EC modification enzymes toward the AM ortholog. While β-lysylation is essential for EF-P activity in EC, subsequent hydroxylation is not [21]. Despite this incomplete modification, AM EF-P still exhibited higher activity with the AM ribosome than EC EF-P did, further supporting the superior functional compatibility between the AM ribosome and its native EF-P.

### Consecutive incorporation of bulky D-α-amino acids using the *A. macleodii* ribosome

**Figure 5.**
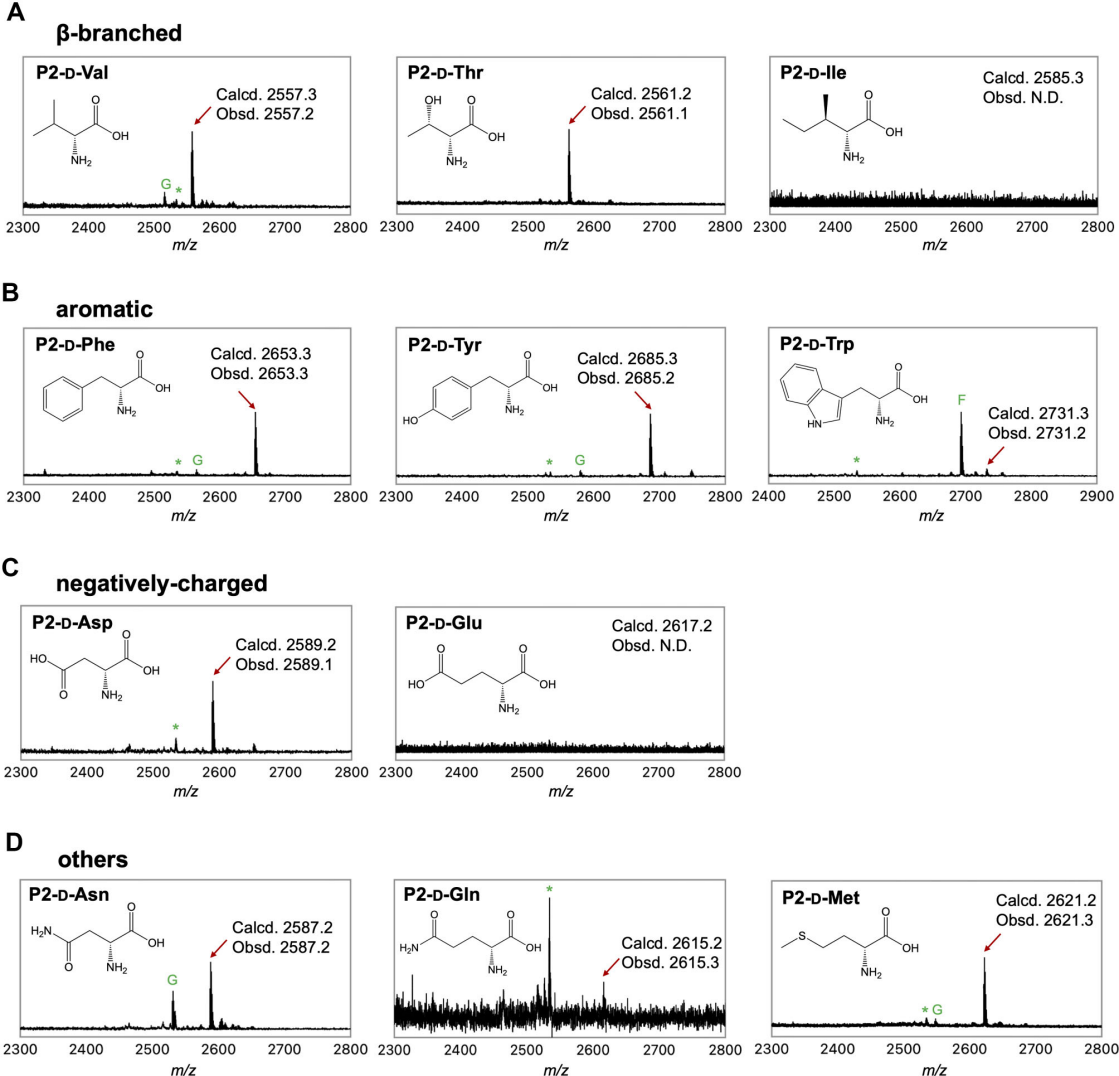
Consecutive incorporation of bulky d-amino acids into P2 using the *A. macleodii* ribosome. (**A**–**D**) MALDI-TOF MS analysis of P2 peptides containing consecutive d-amino acids. Translation was performed with *A. macleodii* (AM) ribosome, along with AM EF-P, AM Uup, and other factors derived from *E. coli*. See Supplementary Table S3 for translation conditions. “Calcd.” and “Obsd.” denote calculated and observed [M + H]^+^ values of the target peptides, respectively. “G” and “F” mark Gly and Phe-misincorporation byproducts. * indicate template-independent impurities originating from the translation system.

While d-Amino acids with small side chains, such as d-Ala and d-Ser, can be consecutively incorporated by the EC ribosome, those with bulkier side chains remain highly challenging substrates. Given that the AM ribosome exhibits greater tolerance toward d-amino acids than the EC ribosome, we hypothesized that the consecutive incorporation of these bulkier residues would be feasible using the AM system. Here, we tested 11 bulky d-amino acids that have not been previously demonstrated for consecutive incorporation (d-Val, d-Thr, d-Ile, d-Phe, d-Tyr, d-Trp, d-Asp, d-Glu, d-Asn, d-Gln, and d-Met). For these challenging substrates, we prioritized MALDI-TOF MS over radiolabeling to verify product identity and purity, as low translation yields and potential misincorporation make precise quantification difficult. Among β-branched d-amino acids, d-Val, d-Thr, and d-Ile were evaluated for their incorporation into P2 using the AM ribosome supplemented with AM EF-P and AM Uup (Fig. 5A). The desired P2-d-Val and P2-d-Thr products were successfully detected by MALDI-TOF MS, although a minor impurity peak resulting from Gly misincorporation was observed for P2-d-Val; in contrast, P2-d-Ile was not detected. The additional methylene group in d-Ile compared to d-Val likely increases the difficulty of its consecutive incorporation. Regarding aromatic d-amino acids, P2-d-Phe and P2-d-Tyr were identified as the primary products, accompanied by small Gly misincorporation peaks (Fig. 5B). Translation of P2-d-Trp proved more challenging, with Phe-misincorporation appearing as the dominant peak, although the target product was still present. Negatively charged d-amino acids, d-Asp and d-Glu, are particularly poor substrates, with even single incorporation being inefficient using the EC ribosome [5, 16]. Notably, the AM ribosome successfully synthesized P2-d-Asp, while P2-d-Glu was not detected (Fig. 5C). The extra methylene group in d-Glu compared to d-Asp further complicates its consecutive incorporation. We also compared d-Asn and d-Gln for incorporation into P2; while both P2-d-Asn and P2-d-Gln were detected, the peak intensity for d-Gln was considerably lower, highlighting the increased difficulty associated with its longer side chain (Fig. 5D). Additionally, consecutive incorporation of d-Met, representing a linear side chain, was successfully achieved (Fig. 5D). In summary, nine out of the eleven tested d-amino acids—d-Val, d-Thr, d-Phe, d-Tyr, d-Trp, d-Asp, d-Asn, d-Gln, and d-Met—were consecutively incorporated for the first time. Although the presence of misincorporation byproducts indicates that these reactions remain inherently difficult, the AM ribosome significantly expands the repertoire of accessible d-amino acids.

### Ribosomal synthesis of a model macrocyclic peptide containing multiple D-amino acids using the AM ribosome

**Figure 6.**
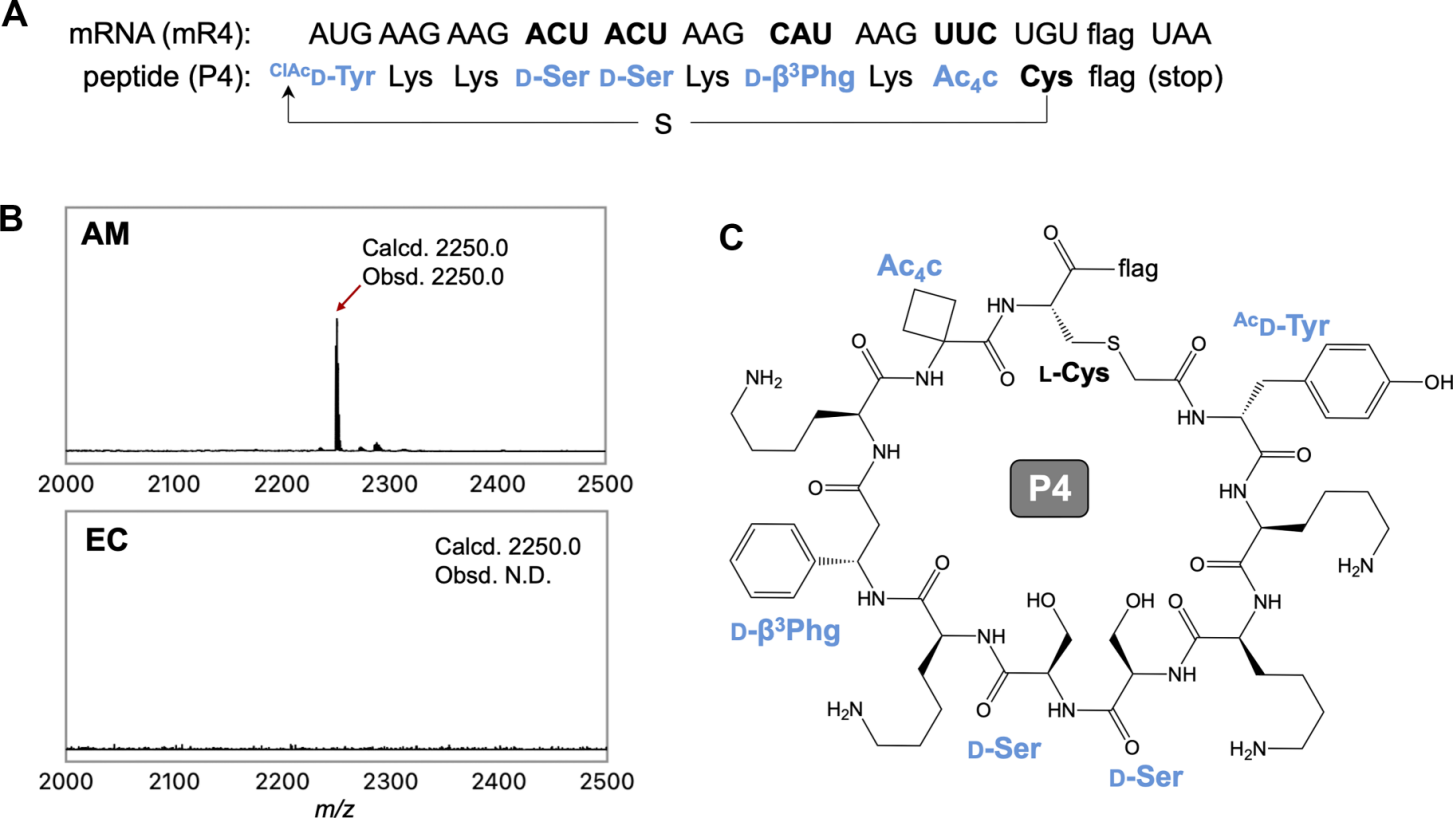
Ribosomal synthesis of a model macrocyclic peptide containing multiple d-amino acids using *A. macleodii* ribosome. (**A**) Sequences of mRNA (mR4) and the corresponding macrocyclic peptide (P4). The “flag” sequence is Asp–Tyr–Lys–Asp–Asp–Asp–Asp–Lys. (**B**) MALDI-TOF MS analysis of translated peptide P4. Translation was performed with either *A. macleodii* (AM) or *E. coli* (EC) ribosomes in the presence of EC or AM EF-P and Uup. See Supplementary Table S3 for details of translation conditions. The arrow marks the monovalent ion ([M + H]^+^) of the desired peptide. “Calcd.” and “Obsd.” indicate calculated and observed [M + H]^+^ values for the peptide. N.D., not detected. (**C**) Chemical structure of P4 peptide. The sulfhydryl group of l-Cys reacts spontaneously with the *N*-terminal ClAc group, forming a thioether bond and a macrocyclic structure.

To further demonstrate the utility of the AM ribosome, we synthesized a model macrocyclic peptide, P4, by simultaneously incorporating three d-α-amino acids, one d-β-amino acid, and one α,α-disubstituted α-amino acid (Fig. 6). Specifically, d-Ser, d-β^3^Phg, and Ac_4_c were precharged onto tRNA^Pro1E2^_GGU_, tRNA^Pro1E2^_GUG_, and tRNA^Pro1E2^_GAA_, respectively, and introduced at the ACU, CAU, and UUC codons of the template mRNA (mR4). Notably, two d-Ser residues were incorporated consecutively.

For macrocyclization, *N*-chloroacetyl-d-tyrosine (^ClAc^d-Tyr) was precharged onto an engineered initiator tRNA (tRNA^iniP^_CAU_, Supplementary Fig. S1B) and introduced at the initiator AUG codon [36]. EF-P recognizes the D-arm motif of tRNA^iniP^, promoting ^ClAc^d-Tyr incorporation. The chloroacetyl group of the *N*-terminal ^ClAc^d-Tyr spontaneously reacts with the thiol group of a downstream Cys to form a stable thioether bond, enabling macrocyclization. As a result, the AM ribosome successfully translated P4, whereas the EC ribosome failed to produce any detectable product (Fig. 6B). This result highlights the significant advantage of the AM ribosome in synthesizing d-amino acid-rich macrocyclic peptides like P4. Remarkably, half of the residues in the resulting macrocycle (5 out of 10) consist of d-amino acids or an α,α-disubstituted amino acid, demonstrating the AM ribosome’s exceptional capacity for the synthesis of highly modified exotic peptides.

## Discussion

In summary, this study demonstrates that the AM ribosome can incorporate a diverse array of d-amino acids and α,α-disubstituted amino acids—including d-β-amino acids—far more effectively than the standard EC ribosome. Notably, the use of the AM ribosome enabled the consecutive incorporation of nine types of bulky d-amino acids for the first time. Although Achenbach *et al.* previously suggested the possibility of consecutive incorporation of these d-α-amino acids using the EC ribosome, their conclusions were based solely on SDS-PAGE analysis without product identification; their results lacked verification by mass spectrometry or peptide sequencing [37]. It is probable that they observed mistranslation byproducts, such as codon skipping or the misincorporation of near-cognate amino acids in place of d-α-amino acids, which are frequently encountered when introducing challenging npAAs [15, 38, 39]. Thus, prior to this work, there has been no direct evidence that these bulky d-amino acids could be consecutively incorporated via ribosomal translation. Our findings provide the first definitive proof of this capability using the AM ribosome.

The AM ribosome-based translation system represents a transformative platform for the discovery of highly modified macrocyclic peptides. In mRNA display-based screening, such as the RaPID system [31, 40], the ability to perform consecutive and multiple incorporations of d-α-, d-β-, and α,α-disubstituted amino acids is not merely an advantage but a prerequisite for library integrity. In a truly random sequence space, successive occurrences of these exotic residues are statistically inevitable; a translation system that fails to navigate such sequences—as the *E. coli* ribosome often does—would suffer from significant library bias, effectively “erasing” vast regions of potentially bioactive chemical space. As demonstrated in Fig. 6, the AM ribosome successfully synthesized a complicated macrocycle where 50% of the residues were exotic, including consecutive d-Ser, a level of modification impossible with the standard *E. coli* system. By ensuring the robust translation of such challenging motifs, the AM-based FIT system allows for the exploration of peptides that incorporate strong turn or helical constraints. For instance, “Aib-d-α-amino acid” segment is a potent turn-inducer that can be used in the design of β-hairpins [41]. The integration of such structural motifs with a macrocyclic scaffold leads to the pre-organization of the resulting peptide into stable, unique conformations, thereby enhancing binding affinity, membrane permeability, and resistance to proteolytic degradation [8, 42]. Consequently, this system is immediately ready for integration into mRNA display workflows, enabling the systematic screening of ‘exotic’ macrocyclic peptides with superior pharmacological profiles that were previously inaccessible through conventional ribosomal synthesis.

**Figure 7.**
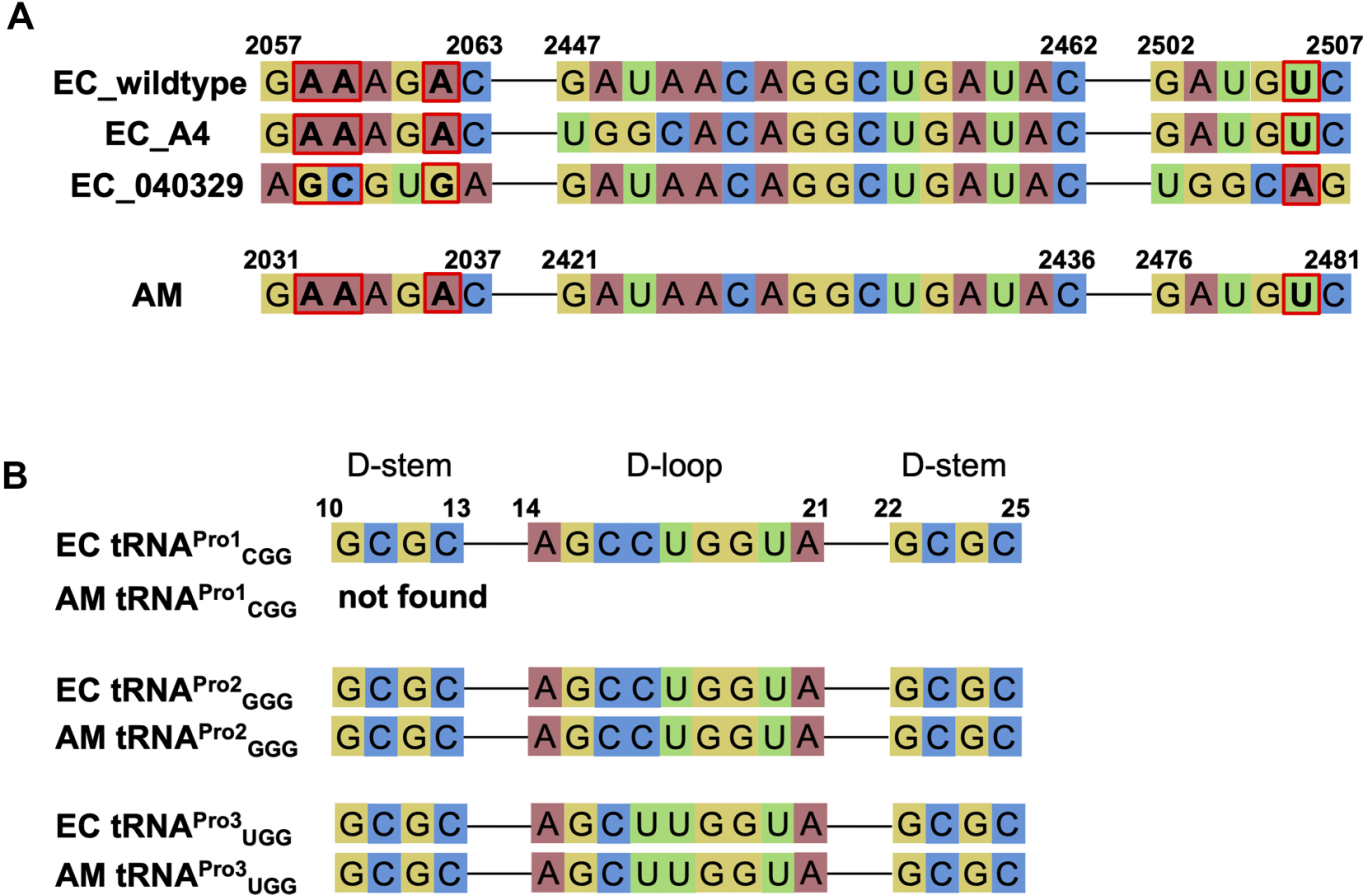
Comparison of the 23S rRNA and tRNA^Pro^ isoacceptors between *E. coli* and *A. macleodii*. (**A**) Sequence comparison of the amino acid recognition site within the PTC of the 23S rRNA. EC_wildtype: *E. coli* K12; EC_A4: mutant *E. coli* ribosome engineered for d-amino acid incorporation [43, 44]; EC_040 329: mutant *E. coli* ribosome engineered for β-amino acid incorporation [49]; AM: *A. macleodii* ATCC 27126. Red boxes indicate the nucleotides implicated in the exclusion of d-amino acids (A2058, A2059, A2062, and U2506 in EC_wildtype; identical to A2032, A2033, A2036, and U2480 in AM). (**B**) Sequence comparison of the D-arms of tRNA^Pro^ isoacceptors between EC and AM.

In 2003, Dedkova *et al.* developed a mutant EC ribosome (A4), featuring mutations in domain V of the 23S rRNA (Fig. 7A, 2447-UGGC-2550), which incorporated d-amino acids more efficiently than the wild-type EC ribosome [43, 44]. While A4 improved stop codon (UAG) suppression with d-Phe and d-Met from 3–5% to 12–23%, these yields remained significantly lower than those for l-Phe and l-Met (52–58%). In contrast, using the AM-based FIT system, the consecutive incorporation of d-Ala into P2 at CCG codons was even more efficient than that of l-Ala (Fig. 2C, 0.23 vs. 0.18 µM). Since the single incorporation of d-Phe and d-Met is relatively efficient even with wild-type ribosomes, the improvements seen with A4 (12–23%) are not particularly remarkable. Fujino *et al.* categorized 19 d-amino acids by single incorporation efficiency [5], with d-Phe and d-Met ranking in the highest group (≥40% yield relative to l-amino acids: Ala, Cys, His, Met, Phe, Ser, Thr, and Tyr). While A4 has not been demonstrated to support consecutive d-amino acid incorporation, the AM ribosome enables thier consecutive incorporation, including multiple d-Phe and d-Met residues. Producing mutant ribosomes like A4 is complicated and labor-intensive, whereas the AM ribosome can be prepared using standard protocols. The AM strain (ATCC 27126) is readily available from the American Type Culture Collection (ATCC) and is easy to culture.

As noted in the Introduction, the conformational changes in A2058, A2059, and A2062 of the 23S rRNA (homologous to A2032, A2033, and A2036 in AM) render the P-site peptidyl-d-Phe-tRNA inactive [30], while U2506 (homologous to U2480 in AM) clashes with the A-site d-Phe-tRNA side chain [19]. Thus, these nucleotide residues—all located within the central loop of the PTC—are considered critical for the exclusion of d-amino acids during translation (Fig. 7A, Supplementary Fig. S7). However, the AM ribosome can incorporate consecutive d-Phe residues into nascent peptides (Fig. 5B), demonstrating its ability to form a peptide bond between P-site peptidyl-d-Phe-tRNA and A-site d-Phe-tRNA despite the bulkiness of the d-Phe side chain. It is possible that the AM ribosome circumvents the involvement of these nucleotide residues in excluding d-amino acids. However, the sequences of the central loop region—including these specific residues (A2058, A2059, A2062, U2506)—are identical between AM and EC (Fig. 7A, Supplementary Fig. S7). Furthermore, regions involved in the recognition and orientation of the tRNA body, such as helices 80, 89, 90, 92, and 93, show no differences between the two species (Supplementary Fig. S7). These observations suggest that the residues in the immediate vicinity of the active center are not the primary cause of the observed differences in d-amino acid incorporation efficiency. On the other hand, in more distal regions, numerous sequence variations exist between EC and AM; the overall sequence identity within domain V is 91.6%. This implies that structural differences in the ribosome as a whole, induced by sequence variations distal to the active center, may influence the overall architecture or the specific orientation of the PTC, thereby enhancing d-amino acid tolerance. It is also possible that differences in ribosomal structural flexibility, rather than just a static “snapshot” of the PTC architecture, contribute to the superior substrate permissiveness of the AM ribosome. Additionally, the EC 23S rRNA possesses nucleotide modifications around the PTC that are involved in regulating translation activity, such as 5-hydroxycytidine at 2501 (ho^5^C2501) and methylations at 5′ carbon of dihydrouridine at 2449 (D^5S^m2449) and 2′-*O*-methylcytidine at 2498 (Cm^5S^m2498) [45]. It is possible that the AM 23S rRNA has a unique and different modification pattern that affects the d-amino acid tolerance, though this remains to be investigated. Future structural analyses of the AM ribosome in complex with peptidyl-d-aminoacyl-tRNA and d-aminoacyl-tRNA are necessary to verify these possibilities.

Kolber *et al.* reported the translation activities of heterologous ribosomes comprising ribosomal proteins of EC and rRNAs of other species, where their activities fell markedly with phylogenetic distance from EC; rRNAs exclusively derived from Gammaproteobacteria and Betaproteobacteria were capable of translation [46]. Their results were comparable to those of this study, where AM (Gammaproteobacteria) and PL (Betaproteobacteria) ribosomes exhibited activities comparable to or higher than the EC ribosome for l-Ala incorporation when paired with EC factors, whereas the ribosomes of phylogenetically more distant species—CC and BS—showed very low or no activities. Thompson *et al.* reported that the *Thermus thermophilus* ribosome, which is also phylogenetically distant from EC, being classified in a different kingdom, Thermotogata, showed only ∼25% translation rate to the EC ribosome in EC S30 extract [47]. These results indicate that the phylogenetic distance between the species is the key parameter affecting the activity of heterologous translation systems and that more homologous ribosome-factor combinations would enhance translation activity due to their structural compatibility. In fact, in P2-d-Ser synthesis, AM-derived EF-P and Uup were more effective than their EC counterparts when paired with the AM ribosome (Fig. 4). EF-P naturally promotes Pro incorporation by recognizing the specific D-arm motif of tRNA^Pro^ isoacceptors [48]. In 2016, we reported that the D-arm motif recognized by EC EF-P consists of a stable 4-bp D-stem with G-C pairs and a 9-nt D-loop [48]. Comparing D-arm sequences, tRNA^Pro2^_GGG_ and tRNA^Pro3^_UGG_ are identical between EC and AM, while tRNA^Pro1^_CGG_ is not present in AM (Fig. 7B). This suggests that AM and EC EF-P recognize the same D-arm motif. Therefore, the EF-P-ribosome compatibility—rather than the EF-P-tRNA compatibility—could be the primary factor driving the enhanced activity in the AM/EC hybrid system.

Looking forward, further improvements could be achieved by replacing all EC-derived factors—including initiation factors, elongation factors, release factors, aminoacyl-tRNA synthetases, and tRNAs—with their AM-derived orthologs. Developing a fully homologous AM-based translation system represents a promising strategy. Moreover, broader screening of diverse bacterial species may identify ribosomes with even higher efficiencies for not only d-amino acids but also *N*-methyl/*N*-alkyl-α-amino acids and β-amino acids, further expanding the horizons of synthetic biology and peptide engineering.

## Supplementary Material

gkag341_Supplemental_Files

## Data Availability

The data underlying this research are available in the article and in its online supplementary material.
