# Supplementary material for: The Alteromonas macleodii ribosome enables consecutive incorporation of bulky D-amino acids into peptides
Source: Nucleic Acids Res. 2026 Apr 21;54(7):gkag341. doi: 10.1093/nar/gkag341 (PMC13096806; doi:10.1093/nar/gkag341)
Supplement: gkag341_Supplemental_Files [file gkag341_supplemental_files.zip › 260320AM_Rbs_supplementary.pdf]

Supplementary Information for:

Alteromonas macleodii ribosome enables consecutive incorporation of bulky D-amino acids into peptides

Takayuki Katoh[1]*, Hiraku Takada[2]*, Maxwell Sigal[1], and Hiroaki Suga[1]

[1]Department of Chemistry, Graduate School of Science, The University of Tokyo, Bunkyo-ku, Tokyo, Japan

[2]Department of Biotechnology, Faculty of Engineering, Toyama Prefectural University, Imizu-shi, Toyama, Japan

*To whom correspondence should be addressed. E-mail: katoh@chem.s.u-tokyo.ac.jp, hirakut58@pu-toyama.ac.jp

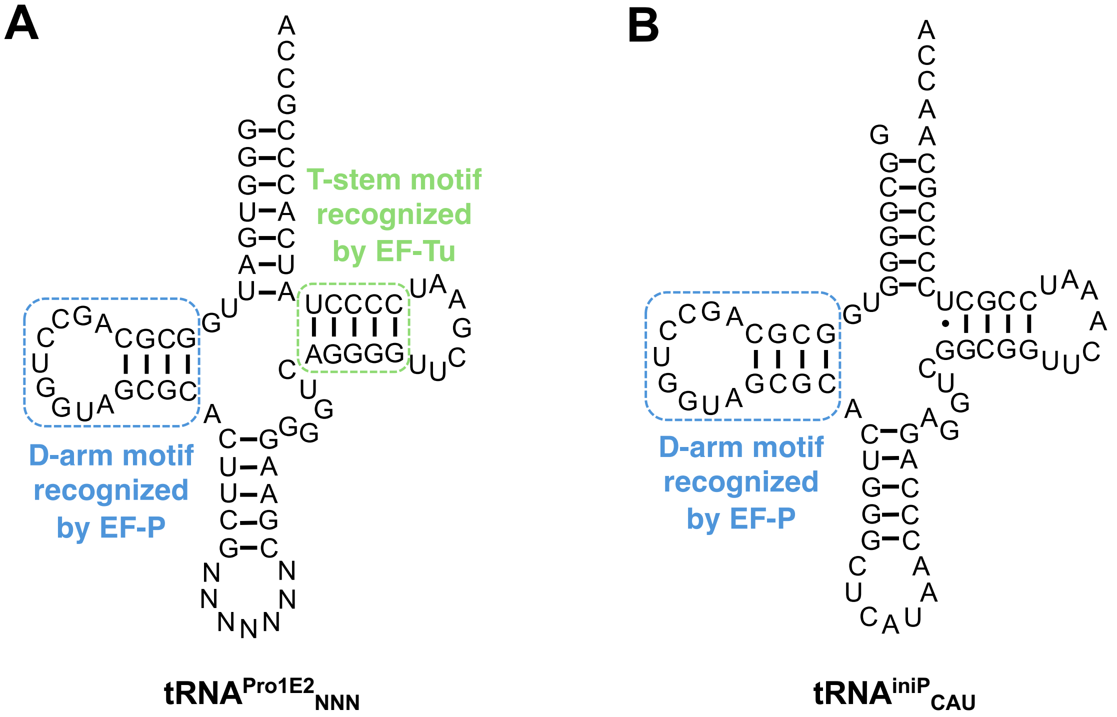

Supplementary Figure S1. Secondary structures of the engineered tRNAs used in this study. (A) tRNA^{Pro1E2} used for npAA incorporation in elongation event. The anticodon loop sequence was changed accordingly to read cognate codons. See Supplementary Table S1 for the anticodon loop sequences. **(B)** tRNA^{iniP} used for ^{ClAc}D-Tyr incorporation in initiation.

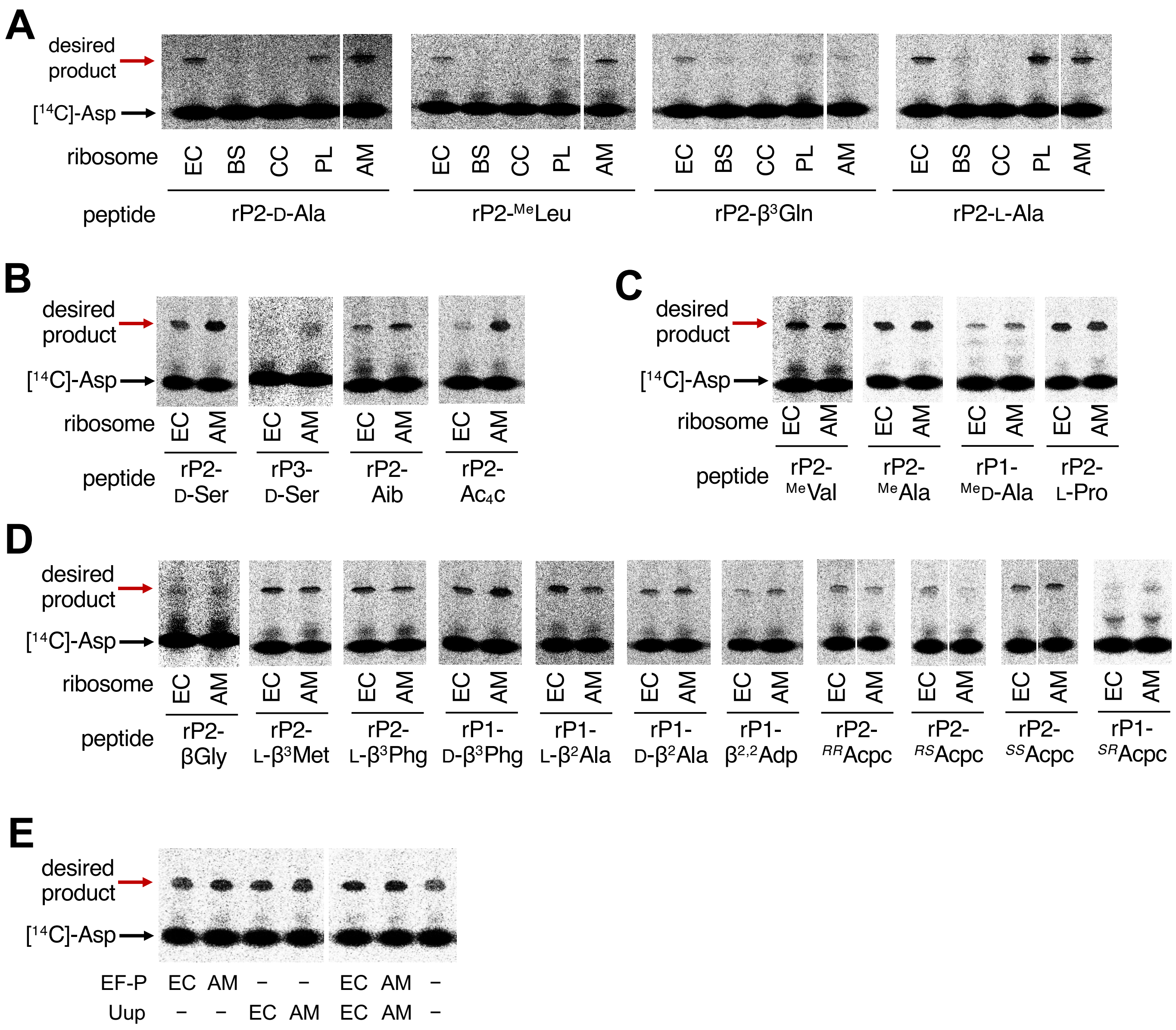

Supplementary Figure S2. Quantification of translated peptides by Tricine SDS-PAGE. (A) Incorporation of D-Ala, ^{Me}L-Leu, L-β^{3}Gln, and L-Ala into peptide P2 using tRNA$^{Pro1E2}_{CGG}$. Translation was performed by using ribosomes of *Escherichia coli* (EC), *Alteromonas macleodii* (AM), *Paraburkholderia largidicola* (PL), *Caulobacter crescentus* (CC), and *Bacillus subtilis* (BS) in combination with *E. coli* translation factors, aminoacyl-tRNA synthetases, tRNAs, and other necessary factors. See also Figure 2C for the quantification results. **(B–D)** Incorporation of D-amino acids and α,α-disubstituted amino acids (B), *N*-methyl/alkyl-α-amino acids (C), and β-amino acids (D) into P1, P2, or P3 using tRNA$^{Pro1E2}_{CGG}$ using *E. coli* (EC) or *A. macleodii* (AM) ribosome. See also Figure 3A–C for the quantification. (**E**) Translation of P2-D-Ser in the presence of *E. coli* or *A. macleodii* EF-P and Uup. Translation was performed using *A. macleodii* (AM) ribosome in combination with translation factors derived from *E. coli*, except for EF-P and Uup. 5 µM EF-P and 1 µM Uup were added to the translation system. See also Figure 4 for the quantification results.

E. coli ribosome

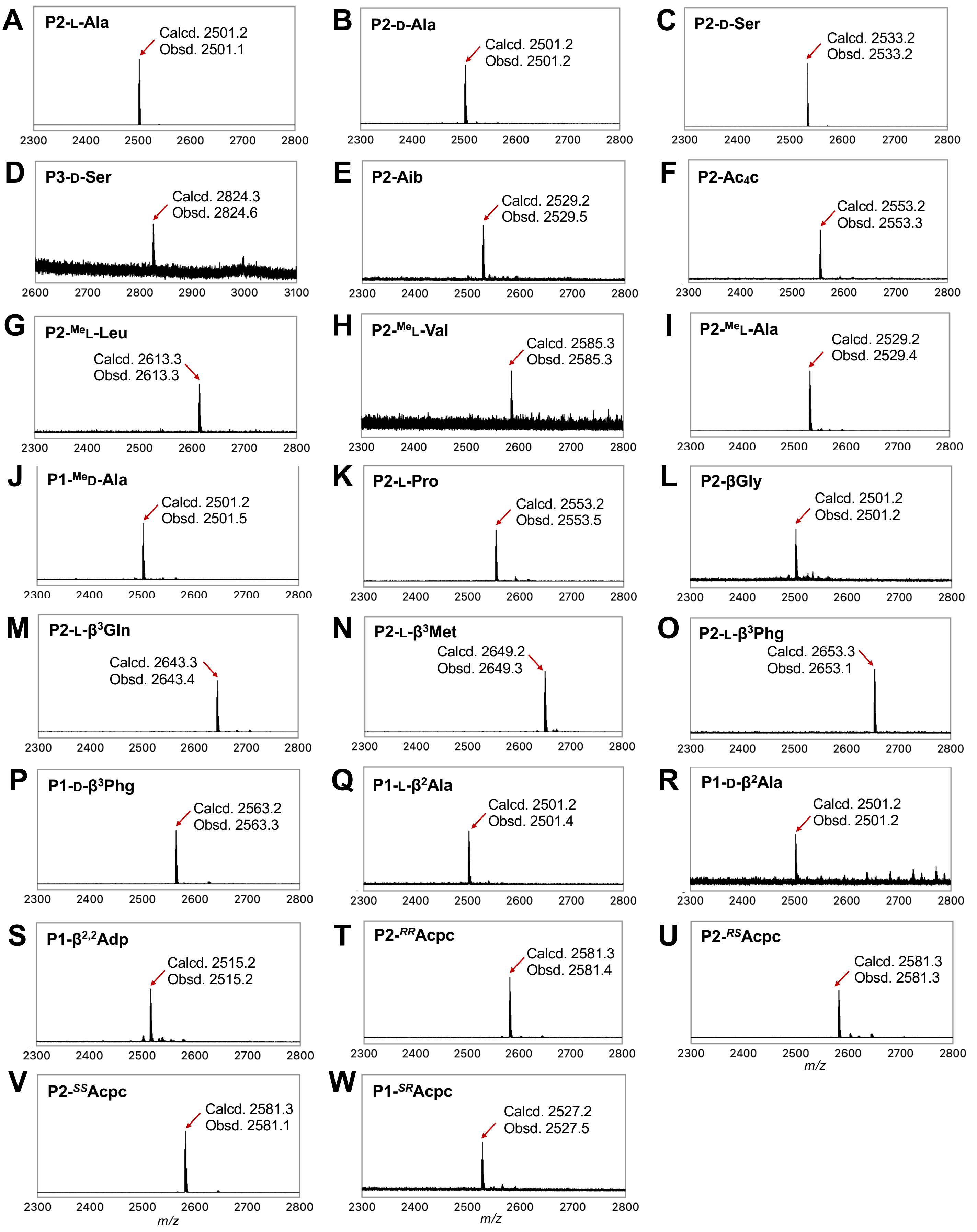

Supplementary Figure S3. MALDI-TOF MS of model peptides translated by *E. coli* ribosome. (A−W) Introduction of L-Ala (A), D-amino acids and α,α-disubstituted amino acids (B−F), *N*-methyl/alkyl-α-amino acids (G−K), and β-amino acids (L−W) into peptide P1, P2, or P3 using $tRNA^{Pro1E2}_{CGG}$. 'Calcd.' and 'Obsd.' indicate calculated and observed $[M+H]^+$ values of the desired peptides, respectively.

A. macleodii ribosome

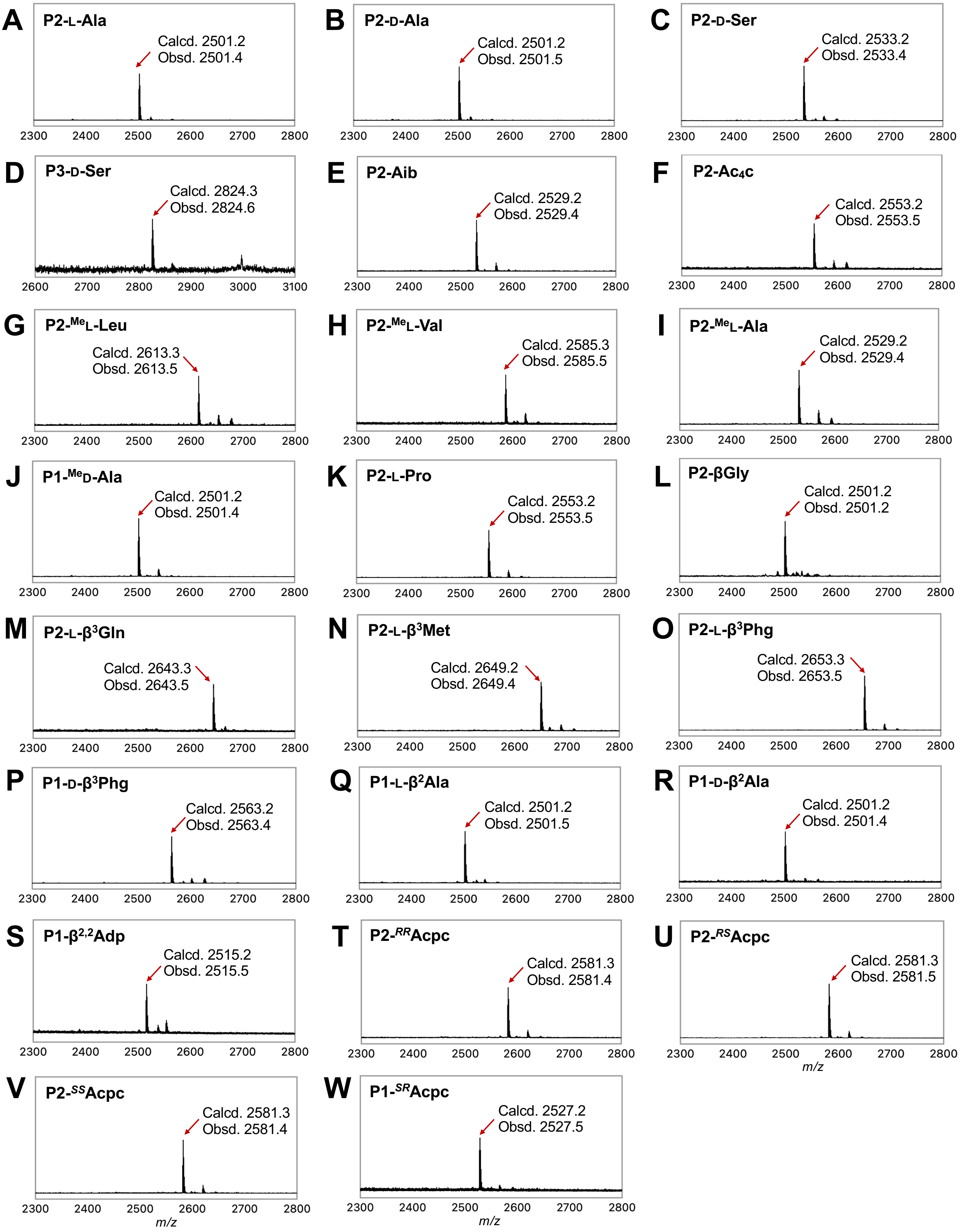

Supplementary Figure S4. MALDI-TOF MS of model peptides translated by *A. macleodii* ribosome. (A−W) Introduction of L-Ala (A), D-amino acids and α,α-disubstituted amino acids (B−F), *N*-methyl/alkyl-α-amino acids (G−K), and β-amino acids (L−W) into peptide P1, P2, or P3 using $tRNA^{Pro1E2}_{CGG}$. ‘Calcd.’ and ‘Obsd.’ indicate calculated and observed $[M+H]^+$ values of the desired peptides, respectively.

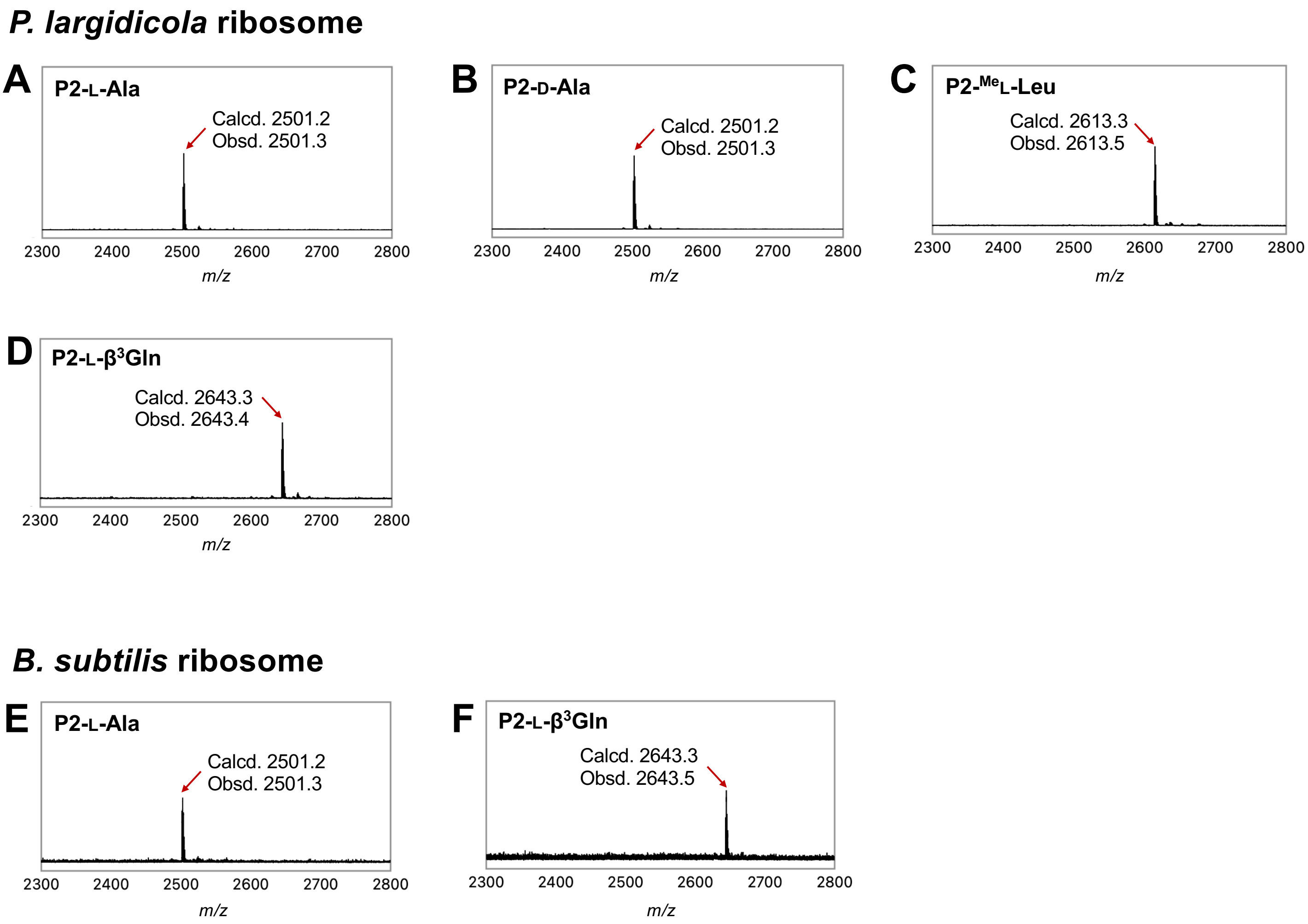

Supplementary Figure S5. MALDI-TOF MS of model peptides translated by *P. largidicola* and *B. subtilis* ribosome. Introduction of L-Ala (A,E), D-Ala (B), ^{Me}L-Leu (C), and L-β^{3}Gln (D,F) into peptide P2 using tRNA$^{Pro1E2}_{CGG}$. 'Calcd.' and 'Obsd.' indicate calculated and observed [M+H]$^{+}$ values of the desired peptides, respectively.

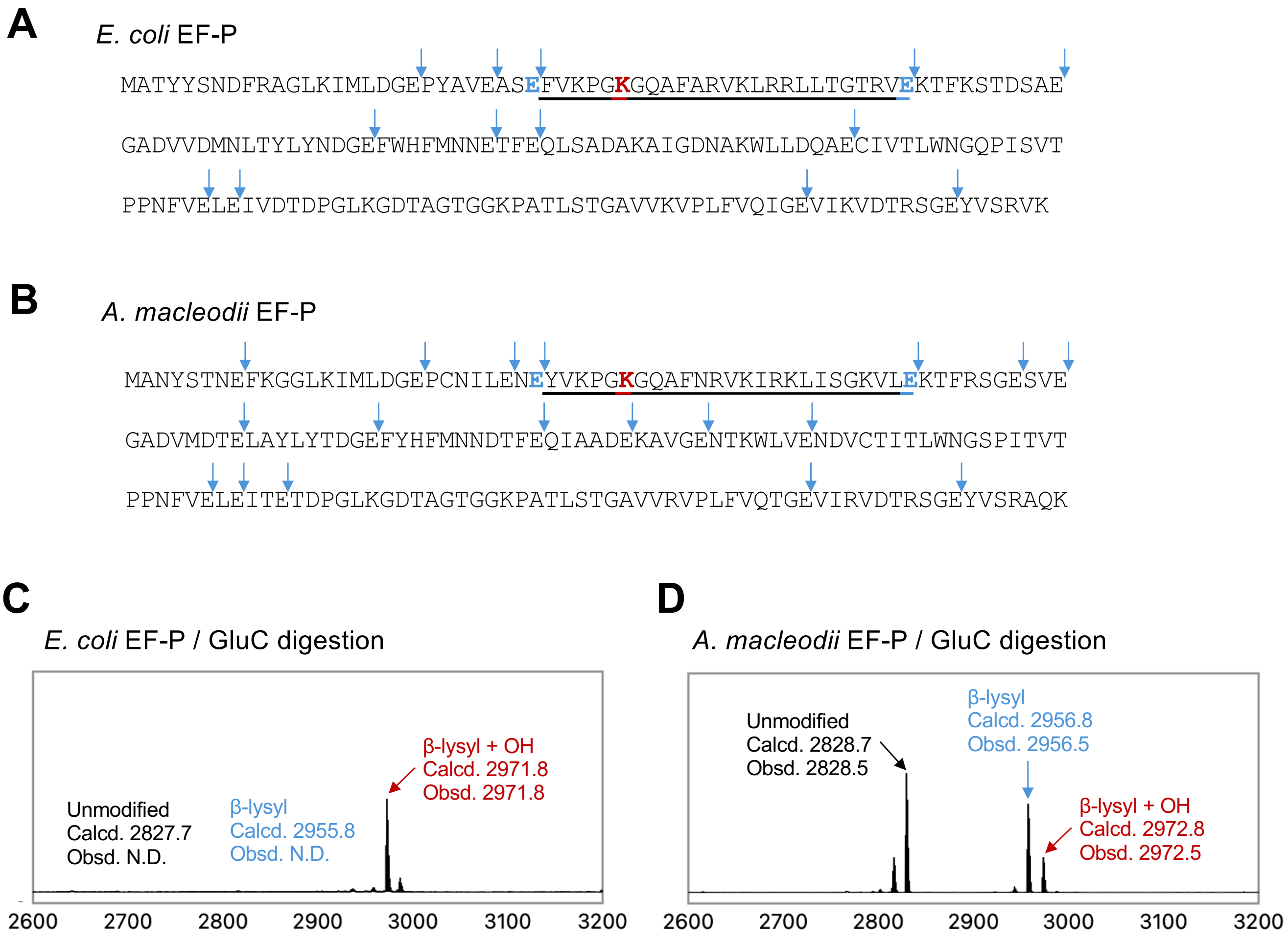

Supplementary Figure S6. Modification of *A. macleodii* EF-P. (**A,B**) Sequences of *E. coli* and *A. macleodii* EF-P. Modified Lys (K34) are indicated by red. GluC digestion sites (C-terminus of Glu) are indicated by blue arrows. The fragments analyzed by MALDI-TOF MS are underlined. (**C,D**) MALDI-TOF MS analysis of EF-P digested with GluC. *E. coli* EF-P (C) and *A. macleodii* EF-P (D). β-Lysylation, β-lysylation+hydroxylation, and unmodified peaks are indicated by blue, red, and black arrows, respectively. ‘Calcd.’ and ‘Obsd.’ indicate calculated and observed $[M+H]^+$ values of the Glu-C digested peptides, respectively. N.D.: Not detected.

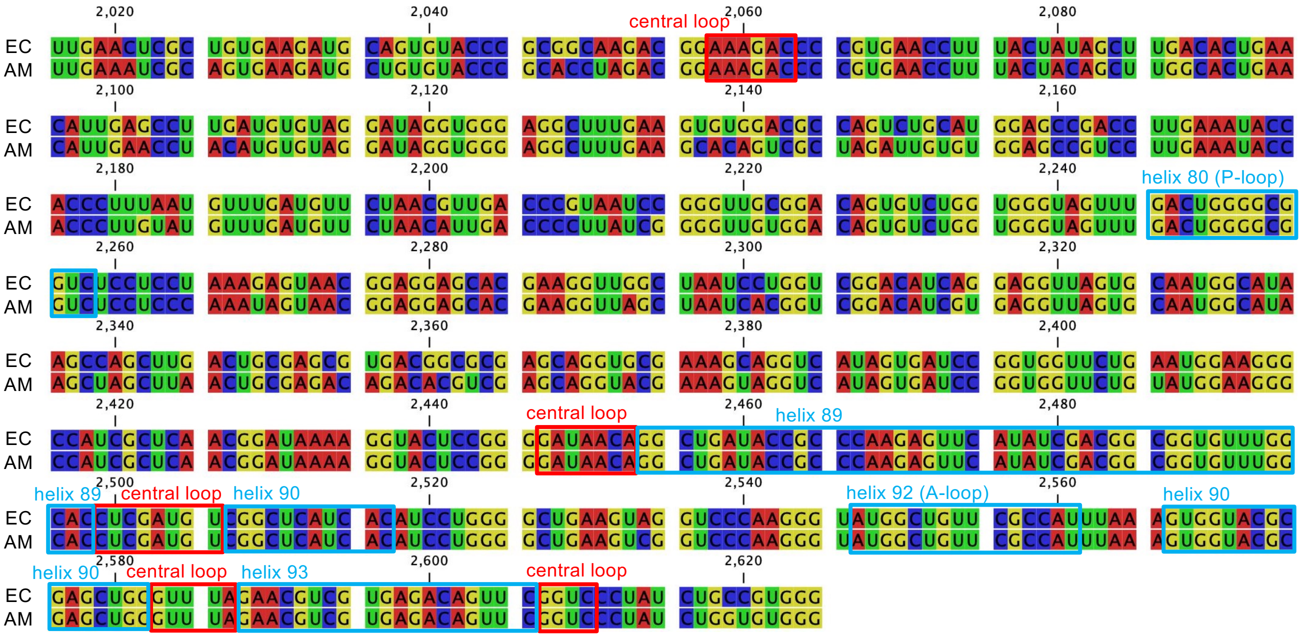

Supplementary Figure S7. Comparison of domain V of the 23S rRNA between *E. coli* and *A. macleodii*. EC: *E. coli* K12; AM: *A. macleodii* ATCC 27126. Red boxes indicate the nucleotides located within the central loop, a region that largely encompasses the amino acid recognition sites shown in Figure 7A. Blue boxes indicate helices 80, 89, 90, 92, and 93, which are involved in tRNA body recognition and orientation within the peptidyl transferase center (PTC). The nucleotide numbering shown is based on the *E. coli* sequence. The sequence identity between EC and AM in this region is 91.6%.
